# Lack of Airborne Transmission during Outbreak of Pandemic (H1N1) 2009 among Tour Group Members, China, June 2009

**DOI:** 10.3201/eid1510.091013

**Published:** 2009-10

**Authors:** Ke Han, Xiaoping Zhu, Fan He, Lunguang Liu, Lijie Zhang, Huilai Ma, Xinyu Tang, Ting Huang, Guang Zeng, Bao-Ping Zhu

**Affiliations:** Chinese Center for Disease Control and Prevention, Beijing, People’s Republic of China (K. Han, F. He, L. Zhang, H. Ma, G. Zeng, B.-P. Zhu); Guangdong Center for Disease Control and Prevention, Guangzhou, People’s Republic of China (K. Han); Zhejiang Center for Disease Control and Prevention, Hangzhou, People’s Republic of China (F. He); Sichuan Center for Disease Control and Prevention, Sichuan, People’s Republic of China (X. Zhu, L. Liu, X. Tang, T. Huang); 1 These authors contributed equally to this article.

**Keywords:** influenza, H1N1, outbreak, pandemic, transmission, droplet, viruses, expedited, China, research

## Abstract

During June 2–8, 2009, an outbreak of influenza A pandemic (H1N1) 2009 occurred among 31 members of a tour group in China. To identify the mode of transmission and risk factors, we conducted a retrospective cohort investigation. The index case-patient was a female tourist from the United States. Secondary cases developed in 9 (30%) tour group members who had talked with the index case-patient and in 1 airline passenger (not a tour group member) who had sat within 2 rows of her. None of the 14 tour group members who had not talked with the index case-patient became ill. This outbreak was apparently caused by droplet transmission during coughing or talking. That airborne transmission was not a factor is supported by lack of secondary cases among fellow bus and air travelers. Our findings highlight the need to prevent transmission by droplets and fomites during a pandemic.

## Abstract 2

This outbreak was caused by droplet transmission.

Since the emergence of a novel influenza A (H1N1) virus (later called influenza A pandemic [H1N1] 2009 virus) in early 2009 in Mexico (*1*,*2*), the virus has spread to 156 countries, territories, and areas; as of July 27, 2009, a total of 134,503 laboratory-confirmed cases and 816 deaths had been reported (*3*). On June 11, 2009, the World Health Organization declared that the world was experiencing the start of the 2009 influenza pandemic (*4*). Investigations of transmission chains early in the pandemic will add to our understanding of the special characteristics of this new virus, including whether its mode of transmission differs from that of seasonal influenza viruses. This information will be useful for effective control of the spread of this virus.

In the People’s Republic of China, the early response strategy has been containment, which includes temperature screening and administration of health questionnaires at international ports of entry, isolation of infected travelers, and quarantine of close contacts of infected persons. During June 2–8, 2009, an outbreak of pandemic (H1N1) 2009 occurred among members of a tour group. We investigated this outbreak to identify the source of infection, mode of transmission, and risk factors for infection.

## Methods

The index case-patient was a 40-year-old female US citizen who had traveled from the United States to Jiuzhaigou, a popular tourist spot in southwestern China; she stopped to change planes in Hong Kong and Chengdu. She noticed her first symptom, chills, on June 2, immediately before arriving in Chengdu, ≈23 hours after departure from the United States. After learning that she had traveled on 3 flights and had toured with a group, we obtained the manifests of all flights that she had traveled on and asked all passengers of the 3 flights and all members of the tour group by telephone or in-person interview whether they had had any symptoms from May 27 through June 12, 2009. We also obtained detailed information on the activities of the tour group during the 3-day tour. Health authorities placed members of the tour group under medical observation and isolated those who had clinical signs or symptoms or positive throat swab culture results. Laboratory technicians at the Sichuan Province Center for Disease Control and Prevention collected throat swabs every 24 hours from all members of the tour group and from symptomatic persons who had shared any of the 3 flights with the index case-patient. Real-time reverse transcription–PCR (RT-PCR) was performed to detect nucleic acids specific for the influenza A pandemic (H1N1) 2009 virus by using the primers supplied by the World Health Organization.

We defined a suspected case as onset of >1 of 5 symptoms—fever (>38°C), cough, sore throat, chills, or headache—in a passenger of flight CZ6659 (June 3, Chengdu to Jiuzhaigou) or flight CZ6660 (June 5, Jiuzhaigou to Chengdu) or in a member of the tour group. A confirmed case was a suspected case for which real-time RT-PCR provided laboratory confirmation of the influenza A pandemic (H1N1) 2009 virus infection. A secondary case was a confirmed case for which the patient’s signs or symptoms began after 9:00 pm on June 3, i.e., at least 24 hours after the onset of the primary (index) case.

To identify the mode of transmission and risk factors for infection, we conducted a retrospective cohort investigation. We interviewed all members of the tour group by telephone or in-person interview to ascertain details of their contact history with the index case-patient.

## Results

**Figure 1:**
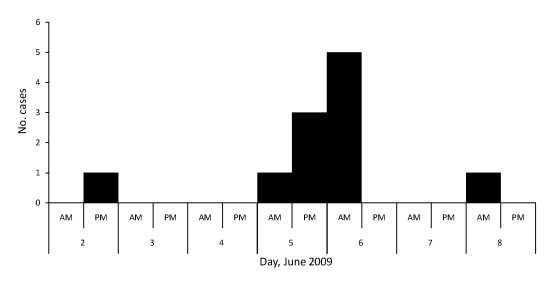
Time of disease onset for persons infected with influenza A pandemic (H1N1) 2009 virus, Sichuan Province, China, June 2009.

During this outbreak, we identified a total of 11 confirmed cases of influenza A pandemic (H1N1) 2009 infection (Figure 1). Average patient age was 36 (range 18–59) years; 2 patients were men and 9 were women. Signs and symptoms were cough (73%), fever (64%), sore throat (64%), headache (27%), chills (27%), runny nose (18%), and myalgia (18%). All 11 case-patients fully recovered; 3 (including the index case-patient) recovered on June 13, 5 on June 15, 1 on June 17, and 2 on June 18. The mean duration of illness was 11 (range 9–14) days.

**Figure 2:**
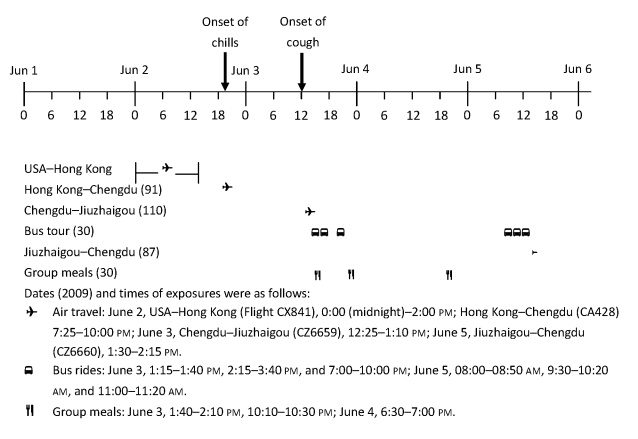
Timeline of exposures to the index case-patient during outbreak of influenza A pandemic (H1N1) 2009, Sichuan Province, China, June 2009. Numbers in parentheses indicate number of persons exposed.

The index case-patient left New York City, United States, on flight CX841 at 12:00 am (midnight) June 2 and arrived in Hong Kong at 2:00 pm on the same day. She transferred to flight CA428 (Boeing 757), which departed Hong Kong at 7:25 pm and arrived in Chengdu at 10:00 pm. On June 3, she and her family members joined the tour group at the Chengdu Airport and boarded flight CZ6659 (Boeing 757), which departed Chengdu at 12:25 pm and arrived at Jiuzhaigou (33°15′55′′N, 104°13′35′′E; average altitude 2,930 m) at 1:10 pm. The group picked up 7 additional members in Jiuzhaigou, where they traveled to various tourist attractions by bus and participated in group activities during the ensuing 3 days. On June 5, the original 24 members of the tour group from Chengdu (without the 7 members who had joined the group in Jiuzhaigou) boarded flight CZ6660 (Boeing 757), which departed Jiuzhaigou at 1:30 pm and arrived in Chengdu at 2:15 pm, along with 87 other passengers (Figure 2).

All airplanes boarded by the index case-patient had high-efficiency particulate air filters. Less than half of the air in the passenger cabins was recirculated; the rest was from outside. Air in the passenger cabins of the airplanes was recirculated approximately every 3 minutes. The air conditioning system in the tour bus mixed ≈70% recirculated inside air with ≈30% outside air, filtered it, and delivered it into the bus through air outlets above the passenger seats. A vent at the back of the bus continually exhausted air from inside the bus.

The diagnosis of influenza A pandemic (H1N1) 2009 virus infection was made for the index case-patient after she returned to Chengdu on June 5. Subsequently, members of the tour group were placed under involuntary medical observation. No influenza-like illness developed in any of the 91 passengers of flight CA428 (Hong Kong to Chengdu) or in any of the 87 passengers on flight CZ6659 (Chengdu to Jiuzhaigou) who were not members of the tour group. None of the passengers on either flight had donned a mask. However, excluding the index case-patient, 9 (30%) of the 30 members of the tour group became ill with secondary cases of disease. The secondary attack rate did not differ between the members of the tour group who flew from Chengdu to Jiuzhaigou and the members who joined the tour in Jiuzhaigou. Of the 87 passengers on the return flight (Jiuzhaigou to Chengdu) on June 5 who were not members of the tour group, 1 person became ill (Table 1). Her seat (9A) was within 2 rows of seats of the index case-patient (7A) and a secondary case-patient (7B), each of whom was symptomatic during the return flight from Jiuzhaigou to Chengdu on June 5.

**Table 1:** Secondary attack rate for influenza A pandemic (H1N1) 2009, by travel history, Sichuan Province, China, June 2009

Group	Total no. persons	No. cases	Attack rate, %
Passengers on flight CA428 (Hong Kong–Chengdu), June 2	91	0	0
Passengers on flight CZ6659 (Chengdu–Jiuzhaigou), June 3	110	7	6.4
Members of the tour group	23	7	30
Not members of the tour group	87	0	0
Members of the tour group, not passengers of flight CZ6659 (Chengdu–Jiuzhaigou), June 3	7	2	29
Passengers on flight CZ6660 (Jiuzhaigou–Chengdu), June 5, not members of the tour group	87	1	1.1

Among members of the tour group, the attack rate was higher for women (50%) than for men (13%) (2-tailed Fisher exact test, p = 0.05). The secondary attack rate among persons 18–39 years of age was 41% (7/17; exact 95% confidence interval [CI] 18–67) compared with 21% (3/14; exact 95% CI 4.7–51) for persons 40–63 years of age.

The index case-patient began having chills at ≈9:00 pm during her flight from Hong Kong to Chengdu. She started coughing before she boarded the flight from Chengdu to Jiuzhaigou on June 3 and continued to cough during the entire tour and after she returned to Chengdu. She had extensive interactions with other members of the group, who talked with each other, helped each other take pictures, gave chewing gum to each other, had group meals together, and stayed in the same hotel. During the 3-day trip, the group traveled together in an air-conditioned tour bus; doors were shut and windows were sealed to conserve energy. While traveling among the various tourist attractions, the group was together on bus rides for a total of 6 hours and 50 minutes. When we evaluated the contact patterns of the tour group with the index case-patient, we found that for the 16 tourists who had talked with the index case-patient from close range (<2 m) for >2 minutes, the attack rate was 56%, whereas none of the 14 tourists who did not talk with her became ill. Members of the tour group who had talked with the index case-patient for >10 minutes were almost 5× as likely to become ill than those who had talked with her for 2–9 minutes (Table 2). The 14 passengers who had not talked with the index case-patient did report other interactions with her, such as dining at the same table, sitting within 2 rows on the same flight or bus ride, and receiving chewing gum from her. Moreover, 3 of these 14 uninfected passengers had sat within 2 seats of the index case-patient during the bus rides but had never talked with her from close range.

**Table 2:** Secondary attack rate of influenza A pandemic (H1N1) 2009 among the tour group members, by exposure, Sichuan Province, China, June 2009*

Exposure	Total no. persons	No. cases	Secondary attack rate, %	Rate ratio (95% CI)
Seat proximity to index case-patient during flight CZ6659, Chengdu–Jiuzhaigou, June 3				
>2 rows	19	5	26	Referent
<2 rows	4	2	50	1.9 (0.18–2.8)
Seat proximity to index case-patient during bus rides				
Never <2 rows	8	2	25	Referent
Ever <2 rows	22	7	32	1.3 (0.35–5.7)
Talked with index case-patient from <2 m for >2 min				
Yes	16	9	56	∞ (2.4–∞)
No	14	0	0	Referent
Length of conversation with index case-patient				
>10 min	10	8	80	4.8 (1.2–70)
2–9 min	6	1	17	Referent

*CI, confidence interval.

## Discussion

Since its emergence, pandemic (H1N1) 2009 has spread around the world, including 1,930 confirmed cases in China as of July 27, 2009 (*5*). Of the cases that have occurred in China (excluding Hong Kong, Macao, and Taiwan), >80% have been imported (*6*); however, several outbreaks caused by transmission from imported case-patients have also occurred in China. Our investigation documented 1 such outbreak.

Seasonal influenza A is transmitted directly by large droplets, or indirectly by fomites (*7*). However, the transmission dynamics of the influenza A pandemic (H1N1) 2009 virus have been less well researched. Our data show that this outbreak was caused by talking with the index case-patient at close range, which indicates droplet transmission. Conversely, other kinds of contact, such as dining at the same table and receiving chewing gum from the index case-patient, played no role during this outbreak.

The role of airborne transmission for influenza is debatable (*7*–*10*). Our investigation did not find evidence of airborne transmission during this outbreak. The lack of cases among 14 tourists who were with the index case-patient in an enclosed bus cabin for nearly 7 hours suggests that airborne transmission was not a factor. The absence of secondary cases among passengers of the flight from Chengdu to Jiuzhaigou also supports this conclusion. Although the case-patient with disease onset on June 8 appeared to have been infected while sharing the flight from Jiuzhaigou to Chengdu, she sat within 2 seats of 2 symptomatic case-patients, which is also consistent with droplet or fomite transmission.

During this outbreak, the index case-patient was febrile while traveling on 3 flights. A secondary case-patient was also febrile while traveling on the return flight (Jiuzhaigou to Chengdu). Neither patient’s illness was detected by thermal scanning at the airports. Another secondary case-patient had had a headache during the return flight. The index case-patient filled out a health questionnaire but did not truthfully inform health authorities of her symptoms. The other 9 case-patients began having symptoms after returning home; hence, they were also not detected by airport screening. These data suggest that thermal scanning and health questionnaires at the airports were not effective for detecting pandemic (H1N1) 2009 infections.

The main limitation of our investigation was the possibility of recall bias; i.e., those who became ill might have more accurately recalled their contact history than those who did not. However, the index case-patient had a highly distinctive hairstyle, which made her easy to remember. Also, interviews about the tourists’ exposure to the index case-patient were conducted within 1 week of the completion of their tour. These 2 factors should have helped minimize any potential recall bias.

In conclusion, this outbreak of influenza A pandemic (H1N1) 2009 virus infection was caused by transmission during coughing or vocalization by an imported case-patient. The virus spread by droplet transmission when the index case-patient was talking with her fellow tourists. The findings of our investigation highlight the importance of preventing droplet transmission during a pandemic.
